# A Novel Wire Is Effective for Echo-Guiding Percutaneous Atrial Septal Defect Closure: A Preclinical Study

**DOI:** 10.1155/2018/5784567

**Published:** 2018-07-03

**Authors:** Yao Liu, Gai-Li Guo, Feng-Wen Zhang, Bin Wen, Wen-Bin Ou-Yang, Yong-quan Xie, Xiang-Bin Pan

**Affiliations:** Department of Cardiovascular Surgery, National Center for Cardiovascular Disease, China & Fuwai Hospital, Chinese Academy of Medical Sciences and Peking Union Medical College, Beijing 100037, China

## Abstract

**Objective:**

To assess the effectiveness of a novel ultrasound wire for echo-guiding percutaneous atrial septal defect (ASD) closure in a sheep model.

**Methods:**

After right lateral thoracotomy, ASDs were created in 20 sheep by transseptal needle puncture followed by balloon dilatation. Animals were evenly randomized into 2 groups to undergo ASD closure using echography as the only imaging tool with either COOK wire (control group) or new ultrasound wire (study group). The total procedural time, passing time (time needed for the guide wire to enter the left atrium), frequency of delivery sheath dropping into the right atrium, frequency of arrhythmias, and 1-week rate of complications were compared between the two groups.

**Results:**

All animals survived defect creation procedures uneventfully. ASD devices were successfully implanted in all sheep. Compared with the control group, the study group had significantly (*P* < 0.05) lower mean procedure time (15.36 ± 4.86 versus 25.82 ± 7.85 min), lower mean passing time (2.69 ± 0.82 versus 5.58 ± 3.34 min), lower frequency of the guide wire dropping into the right atrium (0% versus 40%), and lower frequency of atrial (4.41 ± 2.61 versus 9.60 ± 3.68) or ventricular premature contractions (0.75 ± 0.36 versus 1.34 ± 0.68), respectively, without serious complications up to one week.

**Conclusion:**

The novel ultrasound specialized guide wire was effective in echo-guiding percutaneous ASD closure.

## 1. Introduction

Percutaneous atrial septal defect (ASD) closure has become the treatment of choice in most clinical presentations of ASD [1]. Since proven effective, transthoracic echocardiography (TTE)-guided percutaneous ASD closure (the PAN procedure) has been performed in an increasing numbers of patients [2–4] avoiding exposure to radiation and use of lead suit protection [5]. However, mastering the PAN procedure requires getting used to guidance by echocardiographic plane-by-plane scanning instead of fluoroscopic 3D image projections onto a 2D plane which facilitates finding the guide wire tip [4, 6]. To overcome the latter learning curve limitation and the fact that available guide wires were designed for fluoroscopic and not for echocardiographic guidance, we designed a novel guide wire with a spindle tip, specialized for ultrasound guidance. We here report our initial animal study on the novel guide wire, which facilitated the percutaneous and nonfluoroscopic PAN procedure for ASD closure.

## 2. Materials and Methods

### 2.1. Animal ASD Model Creation

Atrial septal defects were created in adult female black-headed mutton sheep (*n*=20; mean body weight, 45 ± 10 kg) by transseptal puncture and subsequent balloon dilation of the interatrial septum. Animals were anesthetized with xylazine 0.4 mg/kg intramuscularly and an intravenous bolus infusion of 10 mg/kg ketamine, underwent endotracheal intubation, and were mechanically ventilated with isoflurane and oxygen.

**Figure 1:**
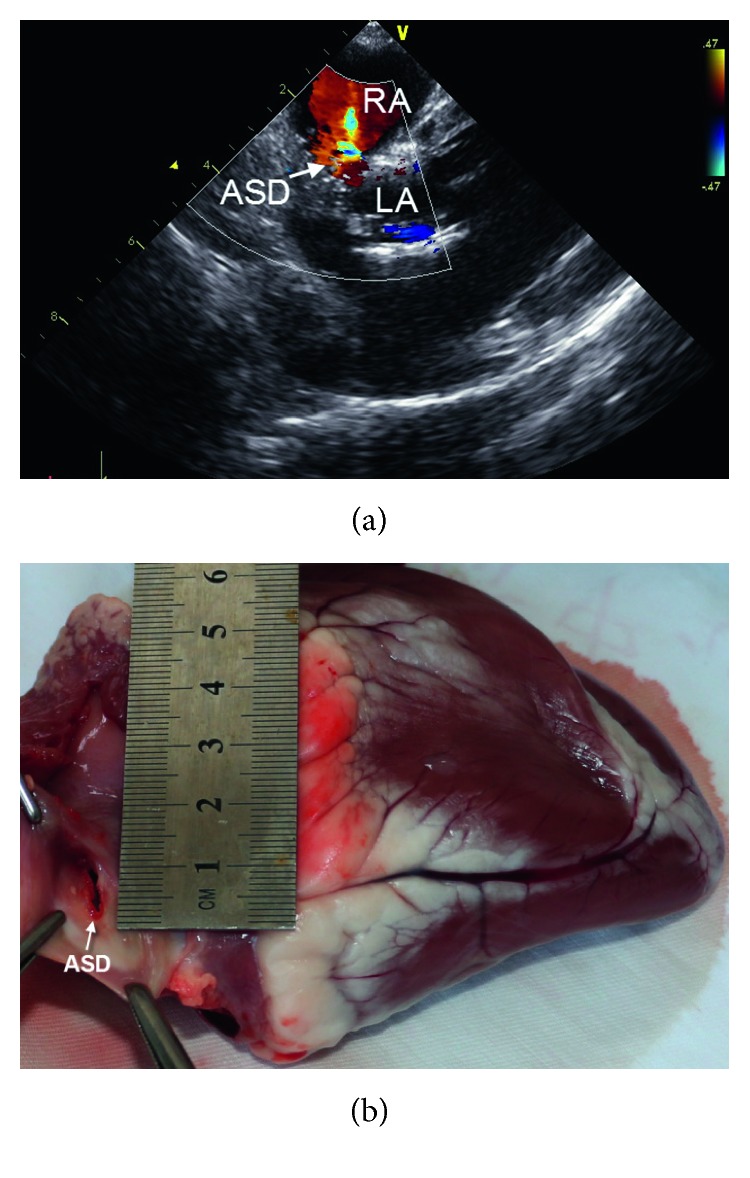
ASD model. (a) Echo imaging of the ASD; (b) gross imaging of the ASD.

The right atrium was exposed via a right midaxillary line incision at the fourth intercostal space level. The pericardium was suspended, and a purse-string suture was placed on the wall of the right atrium. An 8 Fr sheath was inserted into the atrium within the purse string. Following transseptal puncture, a defect within the interatrial septum was created by its subsequent dilation with a 12 mm in diameter balloon (Balt CBV, Montmorency, France) over a guide wire inserted into the left atrium under echocardiography guidance (Figure 1). Heparin (200 U/kg after transseptal puncture) and antibiotics (flucloxacillin 1.5 g at the start of the procedure) were administered intravenously.

The study was conducted in accordance with the Helsinki Declaration for Scientific Experimentation on Animals and approved by the Institutional Animal Care and Use Committee of Fuwai Hospital. All experiments were carried out at the premises of the Department of Experimental Surgery, Fuwai Hospital.

### 2.2. The Novel Ultrasound Guide Wire

**Figure 2:**
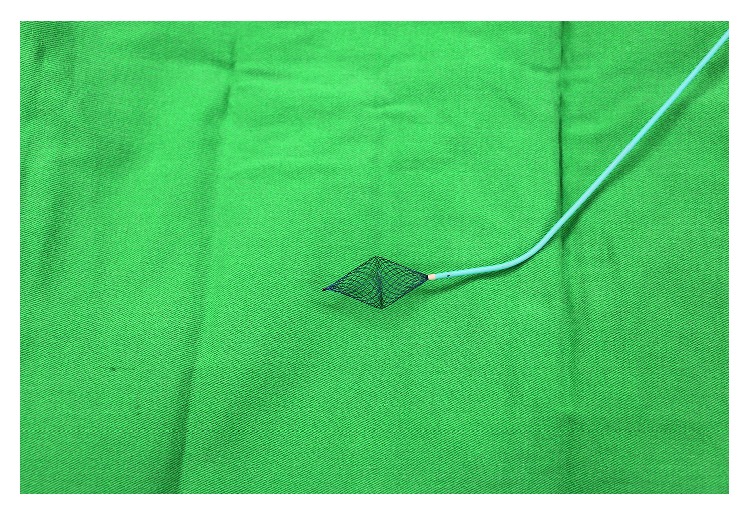
The novel ultrasound guide wire and MPA2 catheter.

**Figure 3:**
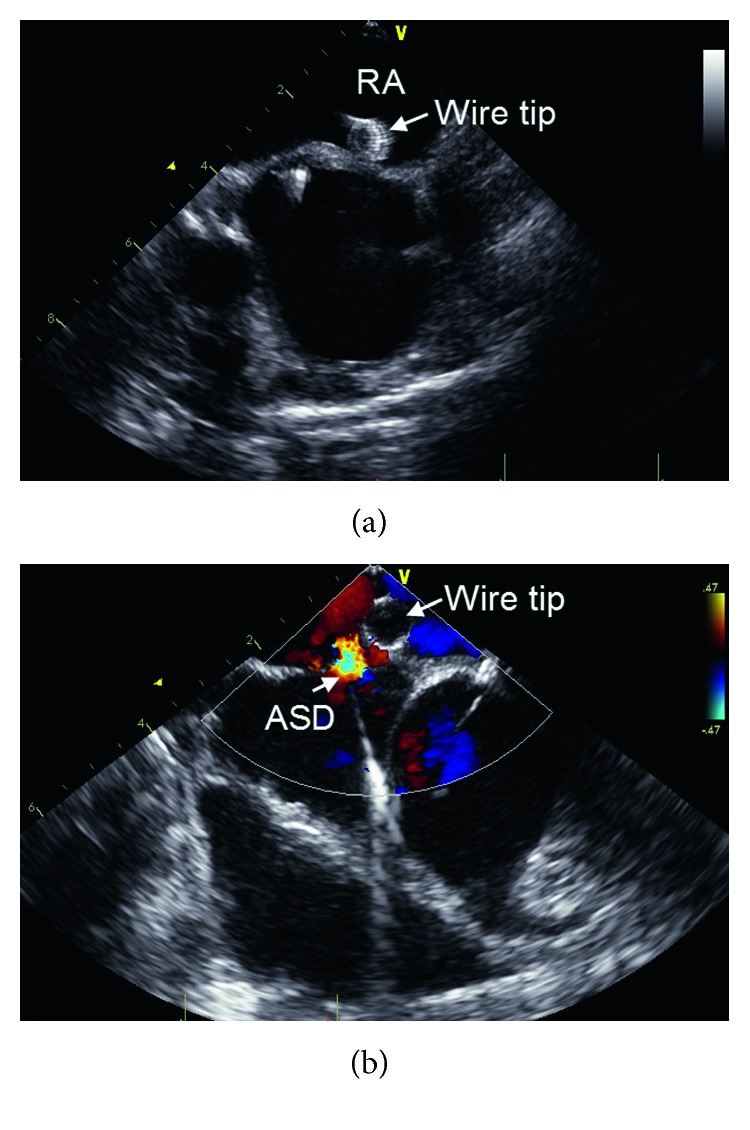
The new wire tip section showed an excellent echogenicity during the procedure. (a) The wire tip in the right atrium (RA); (b) the wire tip and ASD.

The novel guide wire, which is 0.89 mm in diameter and 260 cm long, is made of a high-strength nickel-titanium material, with a nickel-titanium alloy mesh at the tip (Figure 2). The spindle-shaped and 8–26 cm wide tip section is placed on the 6F MPA2 catheter and is easily detected under ultrasound during the procedure (Figure 3). Choosing a guide wire wider than the ASD diameter lowers the rate of guide wire or delivery system dropping into the right atrium. The nickel-titanium alloy mesh prevents puncture damage by deformation when encountering resistance.

### 2.3. Device Implantation and Evaluation

After ASD generation, animals were randomized into 2 groups (10 sheep/group) for ASD closure using echography as the only imaging modality : traditional guide wire group (control group) and new ultrasound guide wire group (study group). Animals were anesthetized as described earlier. The right femoral vein was punctured, and a 6F sheath was inserted. In the study group, the novel ultrasound guide wire with tip width 4 mm larger than ASD diameter was used. The guide wire was loaded into the 6F MPA2 catheter which was then inserted via the 6F sheath into the right atrium, and passed through the defect under subcostal and four-chamber view guidance. An occluder 6–8 mm larger than ASD diameter was then deployed. The control group underwent the same procedure using a lunderquist wire (Cook Medical Inc., USA).

Factors including procedural success rate, passing time, procedure time, number of times the guide wire or delivery system dropped into the right atrium, frequency of arrhythmia, number of times the guide wire traversed tricuspid valve, cardiac perforation, pericardial effusion, and peripheral vascular complications were analyzed to assess the safety and efficacy of the new guide wire. Procedural success was defined as successful implantation of the closure device without embolization or malposition. Procedure time was defined as the interval from 6F MPA2 insertion into the femoral vein to occluder implantation. Passing time was defined as that needed for the guide wire to enter the left atrium.

### 2.4. Statistical Analysis

Statistical analysis was conducted using SPSS 16.0 for Windows (SPSS, Chicago, IL). Continuous variables were expressed as mean ± SD and were compared using two-tailed unpaired Student's *t*-test, while categorical variables were expressed as number (percentage) and were compared using the chi-squared test. *P* < 0.05 was considered statistically significant.

## 3. Results

All animals survived defect generation procedures uneventfully. Man-made ASDs was measured between 6 and 11 mm by two-dimensional echo. Mean ASD diameter (*P*=0.661) and left ventricular ejection fraction (*P*=0.374) were similar between the groups (Table 1).

The ASD devices were successfully implanted in all sheep. As shown in Table 2, compared with the control group, the study group had significantly (*P* < 0.05) lower mean procedure time (15.36 ± 4.86 versus 25.82 ± 7.85 min), lower mean passing time (2.69 ± 0.82 versus 5.58 ± 3.34 min), lower frequency of the guide wire dropping into the right atrium (0% versus 40%), and lower frequency of atrial (4.41 ± 2.61 versus 9.60 ± 3.68) or ventricular premature contractions (0.75 ± 0.36 versus 1.34 ± 0.68), respectively. In both groups, there were no severe complications such as cardiac perforation and peripheral vascular complications. However, in the control group, the guide wire traversed the tricuspid valve in 2 cases and pericardial effusion developed in 1 case.

## 4. Discussion

Since King et al reported atrial septal defect occlusion in 1976, the technology had been widely used due to its minimally invasive advantages, almost ten thousand cases of atrial septal defect occluder were implanted in China each year and still maintained a rapid growth [1, 4, 7]. To avoid fluoroscopy hurts and protect patients and doctors, Pan et al. reported atrial septal defect occlusion under TTE guidance only in 2015 [4, 8]. Even if some experts believed fluoroscopy was not harmful, they also used lead suit to protect themselves, that was the real world [5]. So the TTE-guided new technology had attracted a lot of attention since then, but the promotion process encountered many difficulties, the working mechanism was different between echo and fluoroscopy, and fluoroscopy projects 3D heart on a 2D plane, so it was easy to know where the end of the wire was, and echo had to scan the heart plane by plane, which made it difficult to find the tip of the catheter, thus resulting in a long learning curve.

All the clinical applied guide wires were designed for radiological guidance, not for ultrasound guidance, so we designed a novel guide wire specialized for ultrasound guidance to improve the PAN procedure. The novel guide wire comprised a body made of a steel wire and a spindle-shaped tip braided by a highly elastic nitinol wire. Compared with the straight tip guide wire, the variable tip size wire could be more easily detected by ultrasound and reduced the chance of guide wire dropped into the right atrium. In addition, the novel guide wire made it easier to determine the depth of delivery sheath insertion, and with its great elasticity, it was not easy to damage the posterior wall of the left atrium because the wire would deform when encountered resistance.

Our design was the first specialized ultrasound intervention equipment aiming to overcome the existing technical difficulties. The spindle-shaped tip of the new wire could be easy to detect under ultrasound which overcame the biggest disadvantage of TTE-guided operation; the average time for the wire to pass the ASD was only 2.69 ± 0.82 minutes which was rather short than that for the normal guide wire. No direct injury to valve and no frequent arrhythmia happened in the study group since it was easy to see the tip of the wire under echo. Rarely did the wire drop into the right atrium since the width of the wire was 4–6 mm larger than the diameter of the ASD. In addition, the novel guide wire facilitated determining the depth of delivery sheath insertion and its great elasticity minimized the chance of damaging or perforating the left atrial posterior wall because the wire would deform when encountering resistance thanks to its soft and flexible nickel-titanium tip. No complications and adverse events occurred in the study group.

This guide wire was the beginning of ultrasound-specific equipment and it had successfully facilitated the percutaneous and nonfluoroscopic procedure. Further clinical studies were warranted to verify the effectiveness of this ultrasound-specific device that had the potential to incentivize the use of ultrasound guidance technology.

## 5. Conclusion

In an animal model, use of a novel ultrasound specialized guide wire was effective and facilitated the percutaneous ASD closure under TTE guidance as the only imaging tool.

## Figures and Tables

**Table 1:** Baseline characteristics of the ASD model in both groups.

Baseline characteristics	Study group (*n*=10)	Control group (*n*=10)
Success rate (%)	100	100
ASD diameter (mm)	9.17 ± 1.65	9.45 ± 1.17
Left ventricular ejection fraction (%)	61.90 ± 3.00	60.60 ± 3.37
Tricuspid regurgitation	—	—
Pericardial effusion	—	—

**Table 2:** Procedural characteristics.

Procedure characteristics	Study group (*n*=10)	Control group (*n*=10)
Total procedure time (min)^#^	15.36 ± 4.86	25.82 ± 7.85
Passing time (min)^#^	2.69 ± 0.82	5.58 ± 3.34
Frequency of the guide wire or delivery system dropping into the right atrium^*∗*^	0/10	4/10
Frequency of the guide wire traversing tricuspid valve	1/10	2/10
Frequency of atrial premature contraction^#^	4.41 ± 2.61	9.60 ± 3.68
Frequency of ventricular premature contraction^#^	0.75 ± 0.36	1.34 ± 0.68
Cardiac perforation	0/10	0/10
Pericardial effusion	0/10	1/10
Device implantation rate (%)	100	100

^*∗*^Chi-squared test, *P* < 0.05; ^#^*t*-test, *P* < 0.05.

## Data Availability

The data used to support the findings of this study are available from the corresponding author upon request.
